# Mosquito long non-coding RNAs are enriched with Transposable Elements

**DOI:** 10.1590/1678-4685-GMB-2021-0215

**Published:** 2022-01-24

**Authors:** Elverson Soares de Melo, Gabriel Luz Wallau

**Affiliations:** 1 Fundação Oswaldo Cruz, Instituto Aggeu Magalhães, Departamento de Entomologia e Núcleo de Bioinformática, Recife, PE, Brazil.

**Keywords:** lncRNAs, transposable elements, mosquitoes, Aedes, Culex

## Abstract

Long non-coding RNAs (lncRNAs) lack coding capacity and mounting evidence suggests that they have a regulatory role in diverse organisms. Most knowledge about lncRNAs comes from studies on vertebrates, including a structural association between lncRNAs and transposable elements (TEs). TE sequences are genomic parasites found in all branches of life and are particularly active and abundant in insect genomes. Here we investigate the contribution of TEs to lncRNA biogenesis in *Aedes albopictus* and *Culex quinquefasciatus*. We found that a large fraction of lncRNA loci co-occurs with TE loci in both species. Around 40% of *A. albopictus* and 52% of *C. quinquefasciatus* lncRNAs show some association with TEs. Most of the lncRNA/TE associations are represented by TE-derived sequences that are expressed as one or all exons of lncRNAs, including five lncRNAs that seem to influence immune-related genes involved in antiviral response. The contribution of TEs to lncRNAs also varies among the different types of TEs. The Gypsi superfamily is particularly enriched in lncRNAs sequences. In sum, this study demonstrates that transposable elements substantially contribute to lncRNAs biogenesis in *A. albopictus* and *C. quinquefasciatus* and may have an impact on regulatory modulation in these species.

Mosquitoes of the genera *Aedes*, *Anopheles,* and *Culex* are vectors of several human pathogens. Many studies sought to investigate a range of biological phenomena in these species, such as insecticide resistance and pathogen infection, resulting in thousands of RNA sequencing datasets available. Some recently published articles used dozens of these datasets to characterize long non-coding RNAs (lncRNA) in mosquitoes (Azlan et al., 2021a,b). Many lncRNAs are derived from genes that resemble protein-coding genes (mRNA producers). A mature lncRNA may be processed like a true protein-coding gene mRNA having a similar half-life, but normally they undergo different mRNA processing steps (Ulitsky and Bartel, 2013). However, unlike protein-coding gene mRNAs, lncRNAs are not capable or have a limited capacity of producing fully functional proteins (Mercer et al., 2009). lncRNAs may contain spurious (not translated) or short Open Reading Frames (ORFs), that is, ORFs that might potentially give rise to short polypeptides (<100 amino acids), but these are commonly absent from the species proteome, or if detected, no function can be inferred or characterized (Ulitsky and Bartel, 2013). These small ORFs may have no functional consequences, but in some cases, they may evolve to a more complex polypeptide giving origin to new genes (Ruiz-Orera et al., 2020). Therefore, lncRNAs are a group of RNA molecules larger than 200 nucleotides, expressed from intergenic, intronic or exonic loci, that lack true coding capacity to produce functional proteins (Ulitsky and Bartel, 2013; Cech and Steitz, 2014; Dhanoa et al., 2018). 

Different studies have shown that lncRNAs impact the specimen phenotype in several ways, mainly influencing gene expression, but also regulating chromosome architecture, recruitment of chromatin modifiers, and modulating chromosomal interactions (Bonasio and Shiekhattar, 2014; Yao et al., 2019). In addition, lncRNA knockdowns or knockout experiments result in morphological and behavioral changes in different organisms (Mattick, 2018). Although our knowledge about lncRNA has advanced, there are still several unanswered questions. One example is: from which genomic structures/loci does lncRNA emerge? Strikingly, results from vertebrates showed an important role of transposable elements (TEs) copies in the emergence of lncRNAs (Kelley and Rinn, 2012; Kapusta et al., 2013; Hezroni et al., 2015). More recently, studies on plants also show that some intronic lncRNAs overlap with TEs (Pedro et al., 2018). Although there are few studies on invertebrates, the TE contribution to lncRNA biogenesis appears to vary widely among insects, from almost no TE sequences overlapping with lncRNA in hymenopterans, to considerable overlap in *Drosophila melanogaster* (Lopez-Ezquerra et al., 2017). A recent publication of our group characterized in detail the contribution of TEs to the genome of mosquitoes (Melo and Wallau, 2020)*.* Here, we leverage such a rich TE dataset to evaluate the TE contribution to lncRNA loci in *Aedes albopictus* and *Culex quinquefasciatus.* We detected a broad contribution of some specific TE superfamilies in these two species' lncRNA profiles. 

In order to investigate the role of TEs in lncRNA biogenesis in *A. albopictus* and *C. quinquefasciatus*, we first updated a previous TE characterization conducted by Melo and Wallau (2020) using the most up-to-date genome assembly of both species (GCF_006496715.1 and GCF_015732765.1, respectively). Briefly, we used TEdenovo to generate putative TE consensus families; next filters were applied to remove ill assembled consensus (Figure S1 shows the entire analysis workflow). TE annotation files were generated using the TEannot pipeline (Flutre et al., 2011) with custom parameters. We only kept annotated TE copies larger than 200 nucleotides, as this is the minimum size of an lncRNA. In addition, decreasing the TE copy size increases the chance of spurious matches or superfamily misassignments. 

We then obtained the available annotation from *A. albopictus* RefSeq assembly GCF_006496715.1 and combined it with the lncRNA dataset annotated by Azlan et al. (2021b) to generate lncRNA coordinates of this species. The *C. quinquefasciatus* annotation dataset from RefSeq assembly VPISU_Cqui (GCF_015732765.1) was used as the lncRNA source, we also took the lncRNA dataset annotated by Azlan *et al.* (2021a) to compare between the current and previous assembly version (Cpip2). Lastly, we used GffCompare (Pertea and Pertea, 2020) to find the overlap degree between TEs and lncRNA coordinates in both species.

We found large variability in lncRNA and TE co-occurrence: I - one or multiple TEs can be inserted in introns or exons of a single lncRNA gene; II - TE sequences can overlap intron/exons junctions of a lncRNA gene, or III - a single or multiexon lncRNA can be entirely encompassed by TE coordinates. We found that 37.88% of all *A. albopictus* lncRNAs genes have some degree of overlap with transposable elements (Figure 1A). While this proportion varies in both versions of *C. quinquefasciatus* assembly, from 32% (Figure S2) in CpipJ2 to 52.24% in VPISU_Cqui (Figure 1B). As the latter was the most recent and showed better contiguity, we followed the analysis focusing on this version. Moreover, it is interesting to note that 10.61% and 17.98% of lncRNAs loci are expressed in the TE neighborhood (maximum distance of 2 kb between a lncRNA locus and a TE copy) in *A. albopictus* and *C. quinquefasciatus* respectively. Although not overlapped with a TE region, the expression of these lncRNAs may be modulated by the surrounding TEs transcriptional activity (Lee et al., 2019).

LncRNAs occurring in introns of coding genes (intronic) and between genes (intergenic) were assessed separately and the difference in TE participation in the biogenesis of these two types of lncRNA was very small. In *A. albopictus*, 39.34% of intronic lncRNAs and 37.05% of intergenic lncRNAs overlaps with TEs. This variation is smaller in *C. quinquefasciatus* where 52.86% and 52.36% of intronic and intergenic lncRNAs co-occurs with TEs, respectively. The similarity in the TE/lncRNAs co-occurrence between intronic and intergenic regions differs substantially from vertebrate lncRNAs, where lncRNAs from intergenic and gene-poor regions are richer in TEs than lncRNAs derived from the intronic region and gene-rich part of the genome (Kapusta et al., 2013).

We also evaluate if the intersection between lncRNA genes and TEs occurs more frequently in introns or exons of lncRNAs. We observed that 77.56% and 85.17% of the lncRNA genes that show some degree of overlap with TEs have at least one exon involved with a TE insertion in *A. albopictus* and *C. quinquefasciatus* respectively. This represents 29.37% and 44.49% of all lncRNA expressed in these species. The high fraction of TEs found in lncRNA exons contrasts with some other insects (Lopez-Ezquerra et al., 2017). Thus, the depletion of TEs in lncRNAs exons, as suggested by previous authors, can not be extrapolated for insects in general. At least, in insect species with a large genome and high density of TEs, the contribution of TEs to the evolution and properties of lncRNA can be as important as for vertebrates. In lncRNA exons, TEs can be directly responsible for the lncRNA activity domains (Johnson and Guigó, 2014). Besides, they can also control the expression of lncRNAs via the PIWI-piRNA pathway since the TE regions within lncRNAs may be a target of piRNAs derived from other TE copies dispersed in the genome (Wang and Lin, 2021).

Interestingly, a significant fraction of TE copies that overlaps with lncRNA genes is derived from LTR retrotransposons. They represent 82,82% and 31,89% of lncRNA exons in *A. albopictus* and *C. quinquefasciatus* species (Figure 1C, D). Among these retrotransposons, the Gypsy superfamily stands out. In *A. albopictus*, Gypsy superfamily alone is responsible for more than half of all TE-derived exons of lncRNA (Figure 1E, Table S1), and almost one-quarter in *C. quinquefasciatus* (Figure 1F, Table S1). Strikingly, the proportion of TE superfamily in lncRNA exons does not represent the proportion that TE superfamilies copies found in the mobilome (Chi-squared test, P-value < 2.2e-16) (Figure S3). Performing an over-representation analysis using the clusterProfiler package (Yu et al., 2012), version 3.10.1, we observed that some TE superfamilies are enriched in lncRNA exons of both species (Figure 2B, D). The elements from the Gypsy superfamily represent again one of the most interesting cases. This superfamily shows the highest geneRatio and lower p-values in over-representation analysis among all TE superfamilies of both species (MITE-like elements are not a superfamily, instead they are heterogeneous elements usually derived from DNA transposons that lost their coding region and are flanked by terminal inverted repeats - TIRs). Gypsy elements represent 53.95% of lncRNA exons that overlap with TEs, but only 20.37% of all TE copies found on *A. albopictus* genome (Figure 2A). This discrepancy increases substantially in *C. quinquefasciatus* where Gypsy represents 22.38% of lncRNA exons, but only 1.69% of TE copies on the genome, a 13-fold difference (Figure 2C). The over-representation of some LTR retrotransposons has been observed in human and mouse, but not in insects, where studies on some species showed that TEs on lncRNA reflect genomic TE landscapes (Lopez-Ezquerra et al., 2017). The predominance of a specific TE type in lncRNAs also varies between species, LTR is the main TE order in lncRNA of *A. albopictus*, as in humans, mice, and insects such as *D. melanogaster* (Kapusta et al., 2013; Lopez-Ezquerra, *et al.*, 2017). On the other hand, DNA transposons are prevalent in lncRNA of *C. quinquefasciatus*, as in other insects as *Tribolium castaneum* and *Sogatella furcifera* (Lopez-Ezquerra *et al.*, 2017; Chang et al., 2020). This suggests that the predominant TE type in lncRNAs is species or genus-specific and cannot be extrapolated to family or higher taxa levels.

**Figure 1 -:**
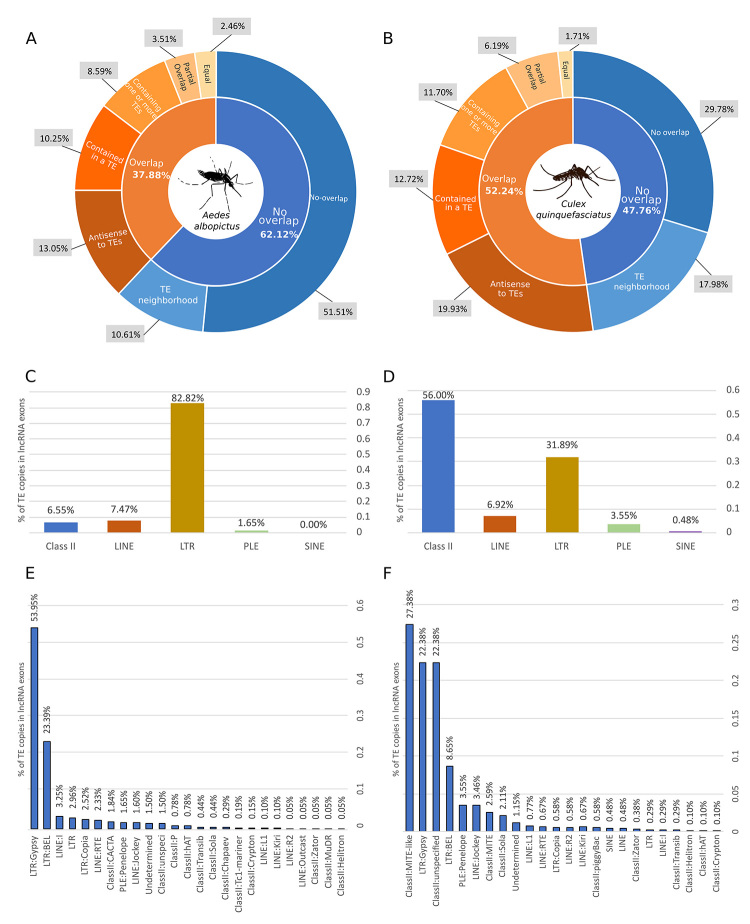
Co-occurrence of lncRNAs and transposable elements in *Aedes albopictus* (A) and *Culex quinquefasciatus* (B). The overlaps were separated into five types: lncRNA overlap with TEs but in the opposite strand; lncRNA contains one or more TEs inserted in their coordinates; lncRNA presents some partial overlap with TEs, in this case, the intersection occurs in 5’ or 3’ of lncRNA; lncRNAs genes are fully derived from a TE copy insertion, where some represent the total length of the TE and other are formed by a specific region of a TE. The proportion of TE-derived lncRNA exons are shown by TE orders in C and D for *A. albopictus* and *C. quinquefasciatus*, respectively. This proportion indicates the prevalence of Gypsy TE in *A. albopictus* (E) and *C. quinquefasciatus* (F).

**Figure 2 -:**
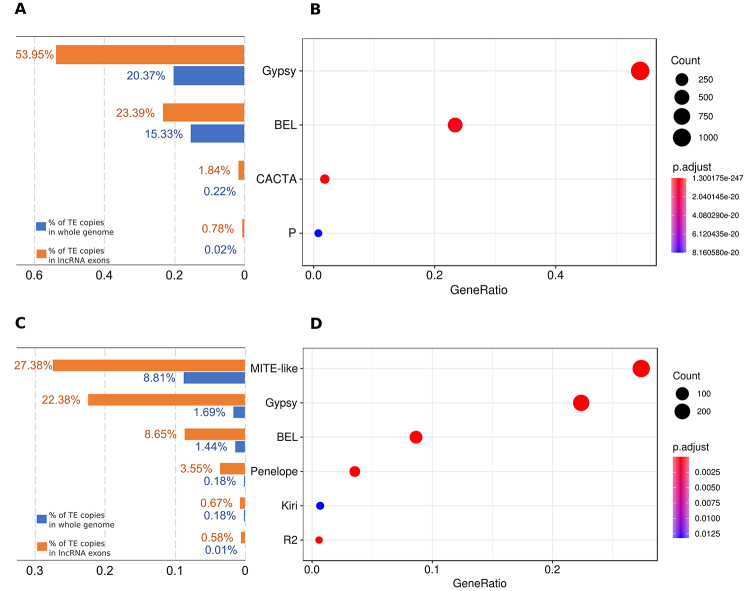
Over-representation analysis of TE superfamilies in lncRNA exons. Distribution of superfamilies that are enriched in *A. albopictus* (A) and *C. quinquefasciatus* (C), a full list of superfamilies is shown in Figure S3. The results of the over-representation analysis show that Gypsy and BEL (LTR retroelements) are the most enriched superfamilies in both *A. albopictus* (B) and *C. quinquefasciatus* (D). MITE-like elements are not a TE superfamily as they are a heterogeneous group of defective elements that have TIRs.

Azlan et al. (2021b) identified 13 lncRNAs that regulate the expression of immune-related genes involved in arbovirus antiviral defense, here we found that five of these lncRNA genes are composed of TEs sequences. Two lncRNAs have one or more TEs inside their introns (XR_003898565.1, XR_003896231.1). The remaining three are fully comprised into TEs copies boundaries, two of them in the same strand (XR_003894180.1, lncRNA_5135.1) and the other on the opposite strand (XR_002127134.2). These three lncRNAs are derived from LTR retroelements (Gypsy, Bel-Pao, and Copia). Moreover, one out of 13 also presents an LTR in its flanking region. LTR retroelements are known to possess long terminal repeats (LTRs) at the boundaries of their coding region. In addition to complete and fragmented LTR retroelements copies, many solo-LTRs are also found dispersed into the genome. LTRs regions contain RNA polymerase II promoters as well as other regulatory sequence motifs, and several studies have been showing that LTRs are more likely to keep these sites than other TE types, increasing the likelihood of modulating flanking genes expression (Chuong et al., 2017). Furthermore, selective pressure on lncRNAs may facilitate the domestication of LTRs as lncRNA promoters (Thompson et al., 2016).

In order to evaluate if there are any distinct TE age patterns of TE copies co-occurring or not with lncRNA exons, the divergence of each TE copy that overlaps with lncRNA and that do not overlap with lncRNAs was estimated against the TE consensus family using blastn (Figure S1). The divergence of each TE copy to the respective TE family consensus sequence represents the relative age of each copy since its split from the ancestral element-the TE family consensus sequence. That is, younger TE copies had less time to accumulate mutations since its split from the ancestral TE and show a lower divergence to the consensus while older TE copies had more time to accumulate mutations and are characterized by a higher divergence to the consensus. A Wilcoxon Rank Sum test (wilcox.test on R) was used to compare the copy divergence between the two datasets. On average, TE copies on lncRNA exons are most similar to TE consensus sequences (younger copies) than TEs copies that are not co-occurring with lncRNAs (Figure 3) for both species. Around 69% and 60% of TE copies involved with lncRNA exons are younger than TE copies dispersed in the genome of the same family, in *A. albopictus* (Figure S4) and *C. quinquefasciatus* (Figure S5) respectively. Such divergence patterns rise two non-mutually exclusive hypotheses: I - TE copies co-occurring with lncRNA are indeed younger insertions; II - such low divergence is a result of conserved old copies that are being maintained under purifying selection. Ulitsky and Bartel (2013) came to the conclusion that lncRNA exons are generally more conserved than intergenic regions (neutrally evolving), in vertebrates (Ulitsky and Bartel, 2013) giving support for the latter hypothesis. However, at the moment, our results are insufficient to differentiate and test these hypotheses due to the lack of orthologous lncRNA loci between *Ae. albopictus* and *C. quinquefasciatus*.

**Figure 3 -:**
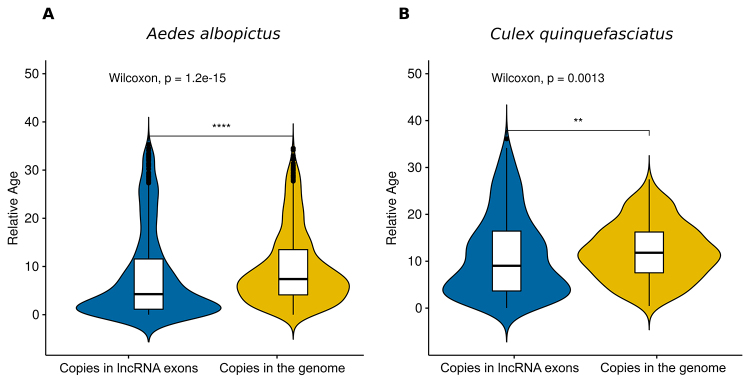
Relative age for TE copies inside (blues) and outside (yellow) lncRNA exons of *Aedes albopictus* (A) and *Culex quinquefasciatus* (B). Relative age was calculated using the blastn divergence of the copies for each TE family against the TE family consensus. ** means a p-value < 0.01, **** means a p-value < 0.0001.

In summary, our study demonstrates that a significant fraction (~37-52%) of long non-coding RNAs from *A. albopictus* and *C. quinquefasciatus* mosquitoes is involved with transposable elements copies. A portion of these TEs may actively contribute to the regulation of the mosquito’s defense against viral pathogens, and probably to other functions in mosquitoes. From this point of view, a fraction of TE copies that gave rise to functional lncRNAs would no longer be considered genomic parasites or selfish elements, but as a host gene that was co-opted for regulatory functions, as shown in previous studies (Chuong et al., 2017). Our study, also shows that there is an over-representation of LTR retrotransposons co-occurring with lncRNAs for both studied species, which differ from previous observations in *Anopheles gambiae*. Although several TE superfamilies are distributed across species (Petersen et al., 2019), co-option for regulatory functions as lncRNAs exons appears to be species or genus-specific, which is supported by the fact that the predominant types of TEs in lncRNAs are different among different insects’ species. Finally, the distribution of TEs in different types of lncRNA, as well as their depletion or enrichment in exons of these structures, does not seem to be characteristic of a group of organisms. Following this reasoning, the profile of TE superfamilies present in lncRNAs is substantially different between species and does not seem to depend on the proportion of superfamilies in the mobilome. However, definitive responses to the influence of TE on lncRNAs for the entire Culicidae family of insects will require additional RNA-Seq experiments from various species belonging to different tribes that compose this taxon.

## Supplementary material

The following online material is available for this article:

Figure S1 - The workflow diagram of the analysis performed in the study.

Figure S2 - Cooccurrence of lncRNAs and transposable elements in the previous version of *C. quinquefasciatus* assembly (CpipJ2).

Figure S3 - Different distribution of TE superfamilies in genome and in lncRNA exons.

Figure S4 - Paired box plot showing the mean divergence of the copies for each TE family against the TE family consensus for *Aedes albopictus*.

Figure S5 - Paired box plot showing the mean divergence of the copies for each TE family against the TE family consensus for *Culex quinquefasciatus*.

Table S1 - The proportion of TE-derived lncRNA exons in both species.
