# Supplementary material for: Mosquito long non-coding RNAs are enriched with Transposable Elements
Source: Genet Mol Biol. 2022 Jan 24;45(1):e20210215. doi: 10.1590/1678-4685-GMB-2021-0215 (PMC8796034; doi:10.1590/1678-4685-GMB-2021-0215)
Supplement: Figure S1 - [file 1415-4757-GMB-45-1-e20210215-s1.pdf]

Genetics and Molecular Biology
https://doi.org/10.1590/1678-4685-GMB-2021-0215

# Supplementary Material to "Mosquito long non-coding RNAs are enriched with Transposable Elements"

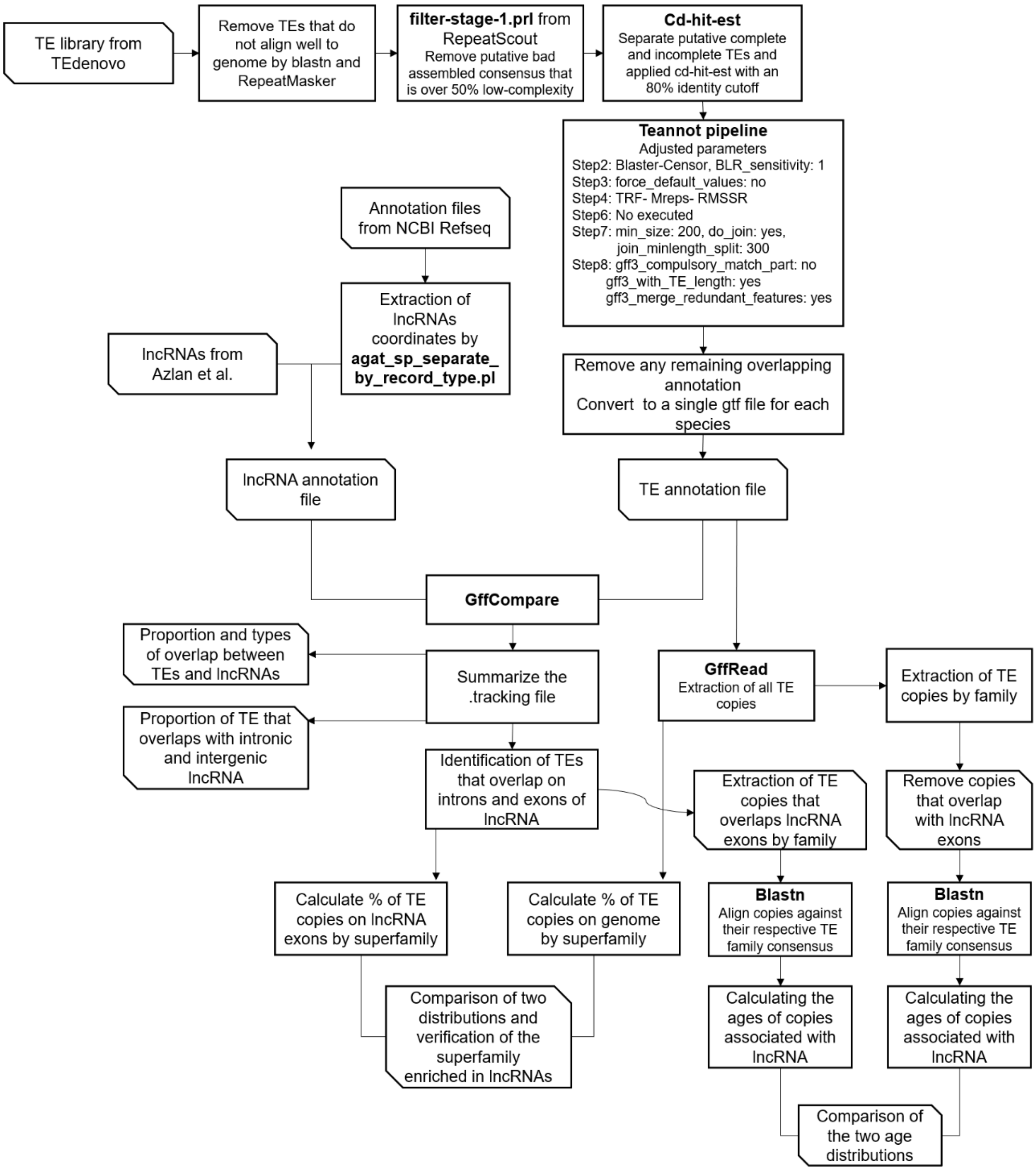


**Figure S1** – The workflow diagram of the analysis performed in the study.
