# Supplementary material for: Mosquito long non-coding RNAs are enriched with Transposable Elements
Source: Genet Mol Biol. 2022 Jan 24;45(1):e20210215. doi: 10.1590/1678-4685-GMB-2021-0215 (PMC8796034; doi:10.1590/1678-4685-GMB-2021-0215)
Supplement: Figure S2 - [file 1415-4757-GMB-45-1-e20210215-s2.pdf]

Genetics and Molecular Biology
https://doi.org/10.1590/1678-4685-GMB-2021-0215

Supplementary Material to "Mosquito long non-coding RNAs are enriched with Transposable Elements"

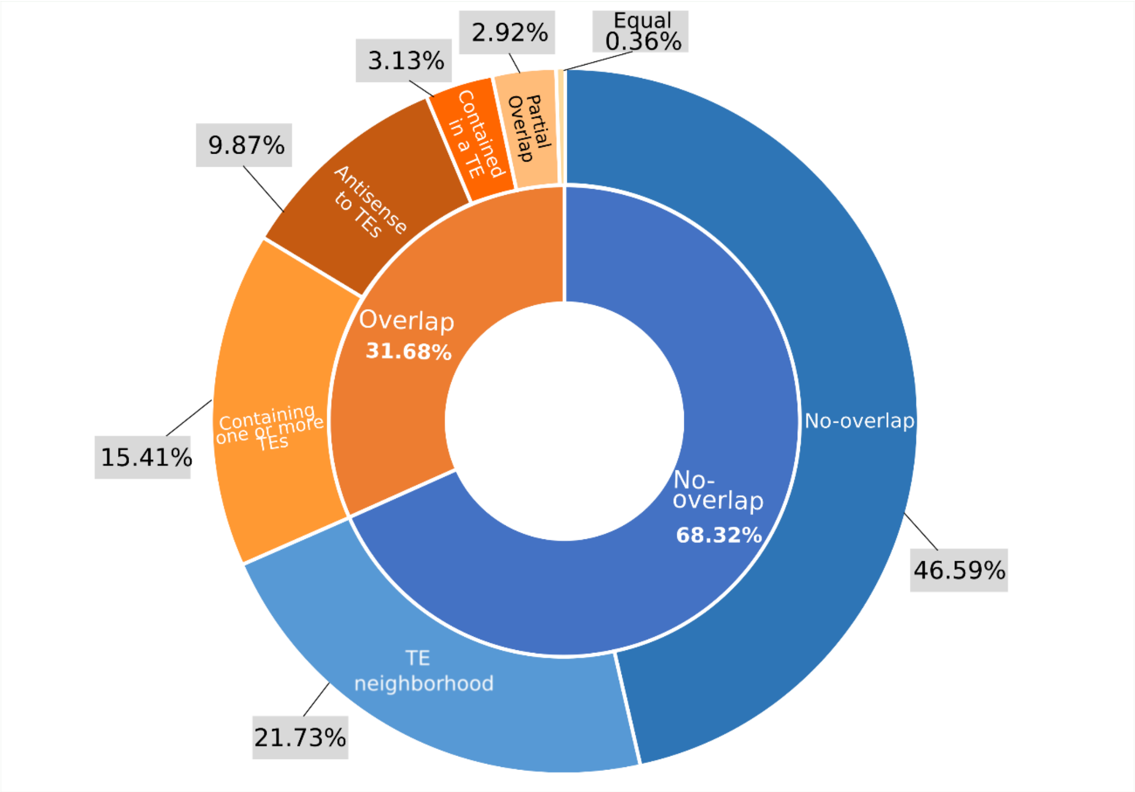

Figure S2 – Cooccurrence of lncRNAs and transposable elements in the previous version of *C. quinquefasciatus* assembly (CpipJ2).
