# Supplementary material for: Mosquito long non-coding RNAs are enriched with Transposable Elements
Source: Genet Mol Biol. 2022 Jan 24;45(1):e20210215. doi: 10.1590/1678-4685-GMB-2021-0215 (PMC8796034; doi:10.1590/1678-4685-GMB-2021-0215)
Supplement: Figure S3 - [file 1415-4757-GMB-45-1-e20210215-s3.pdf]

Genetics and Molecular Biology
https://doi.org/10.1590/1678-4685-GMB-2021-0215

Supplementary Material to “Mosquito long non-coding RNAs are enriched with Transposable Elements”

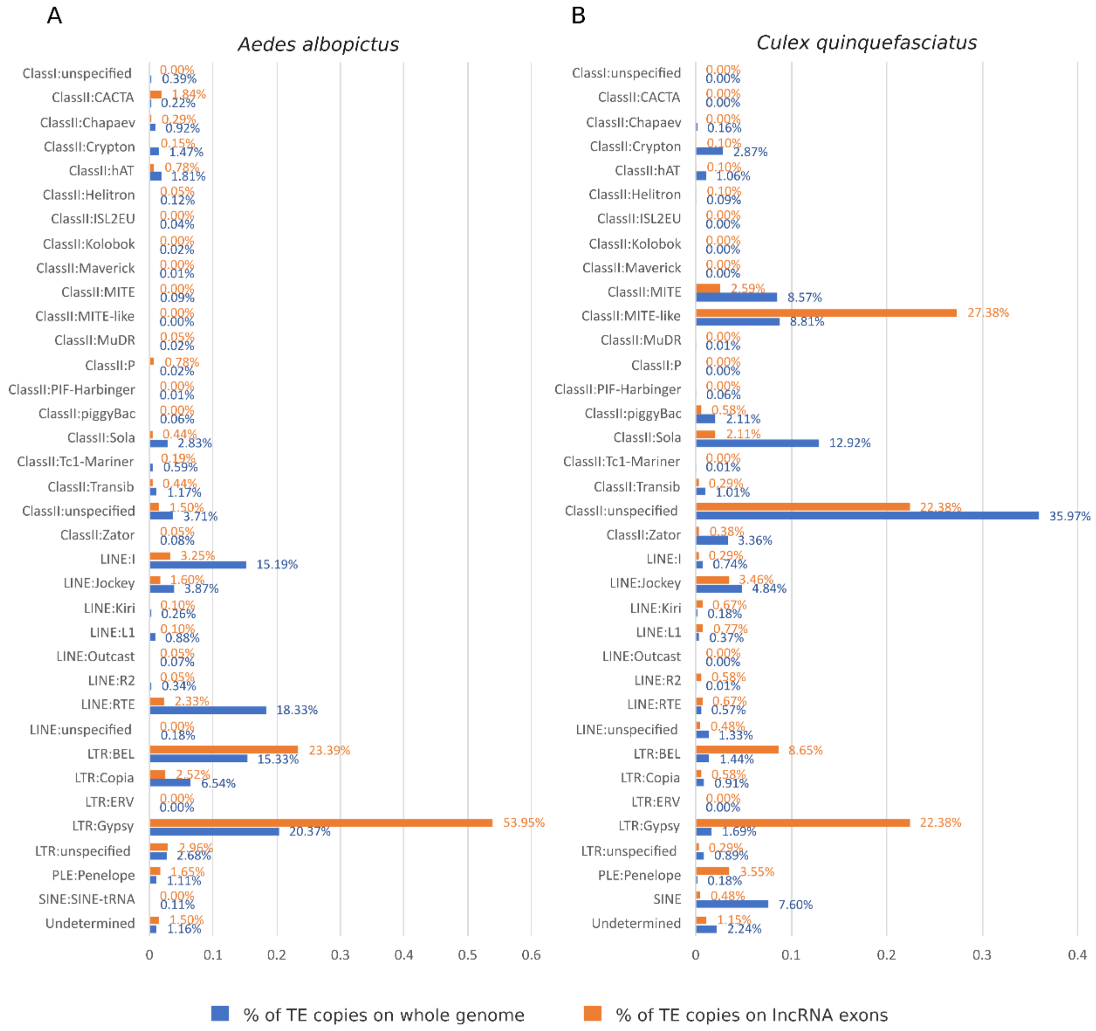

Figure S3 – Different distribution of TE superfamilies in genome and in lncRNA exons. Orange bars represent the fraction of TE copies that overlaps with lncRNA exons by each TE superfamily (Nº of copies by each superfamily in lncRNA exons/Total Nº of copies from that overlap with lncRNA exons), blue bars represent the fraction of all genomic TE copies by each superfamily (Number of copies by each superfamily/Total number of copies from the mobilome).
