# Supplementary material for: Mosquito long non-coding RNAs are enriched with Transposable Elements
Source: Genet Mol Biol. 2022 Jan 24;45(1):e20210215. doi: 10.1590/1678-4685-GMB-2021-0215 (PMC8796034; doi:10.1590/1678-4685-GMB-2021-0215)
Supplement: Figure S4 - [file 1415-4757-GMB-45-1-e20210215-s4.pdf]

Genetics and Molecular Biology
https://doi.org/10.1590/1678-4685-GMB-2021-0215

Supplementary Material to "Mosquito long non-coding RNAs are enriched with Transposable Elements"

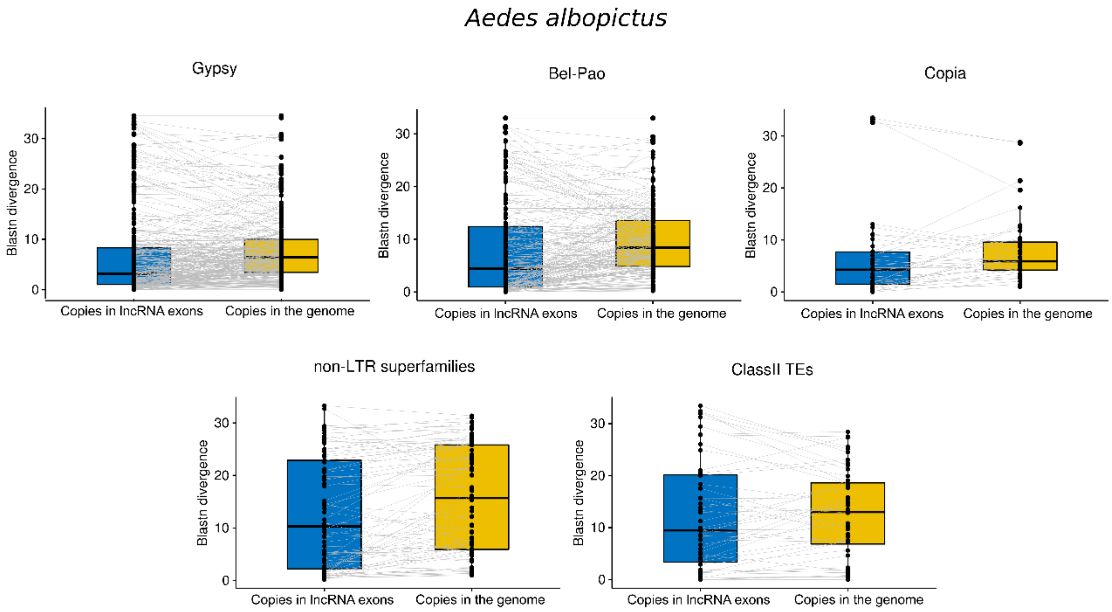

Figure S4 – Paired box plot showing the mean divergence of the copies for each TE family against the TE family consensus for *Aedes albopictus*. Gray lines connect the divergence of the same family calculated using only copies inside lncRNA exons (blue box) and all TE family insertions on the genome (yellow box).
