# Supplementary material for: Mosquito long non-coding RNAs are enriched with Transposable Elements
Source: Genet Mol Biol. 2022 Jan 24;45(1):e20210215. doi: 10.1590/1678-4685-GMB-2021-0215 (PMC8796034; doi:10.1590/1678-4685-GMB-2021-0215)
Supplement: Table S1 - [file 1415-4757-GMB-45-1-e20210215-s6.pdf]

Genetics and Molecular Biology
https://doi.org/10.1590/1678-4685-GMB-2021-0215

Supplementary Material to "Mosquito long non-coding RNAs are enriched with Transposable Elements"

Table S1 - The proportion of TE-derived lncRNA exons in both species.

Superfamily	% of TE copies in lncRNA exons *Culex quinquefasciatus*	% of TE copies in lncRNA exons *Aedes albopictus*
ClassII:Undetermined	22.382%	1.504%
ClassII:CACTA	0.000%	1.844%
ClassII:Chapaev	0.000%	0.291%
ClassII:Crypton	0.096%	0.146%
ClassII:hAT	0.096%	0.776%
ClassII:Helitron	0.096%	0.049%
ClassII:MITE	2.594%	0.000%
ClassII:MITE-like	27.378%	0.000%
ClassII:piggyBac	0.576%	0.000%
ClassII:MuDR	0.000%	0.049%
ClassII:P	0.000%	0.776%
ClassII:Sola	2.113%	0.437%
ClassII:Tc1-Mariner	0.000%	0.194%
ClassII:Transib	0.288%	0.437%
ClassII:Zator	0.384%	0.049%
LINE	0.480%	0.000%
LINE:I	0.288%	3.251%
LINE:Jockey	3.458%	1.601%
LINE:Kiri	0.672%	0.097%
LINE:L1	0.768%	0.097%
LINE:Outcast	0.000%	0.049%
LINE:R2	0.576%	0.049%
LINE:RTE	0.672%	2.329%
LTR	0.288%	2.960%
LTR:BEL	8.646%	23.387%
LTR:Copia	0.576%	2.523%
LTR:Gypsy	22.382%	53.954%
PLE:Penelope	3.554%	1.650%
SINE	0.480%	0.000%
Undetermined	1.153%	1.504%
